# Diabetes and Cancer: The Perfect Storm and a PRICE to Pay

**DOI:** 10.3390/cancers16122247

**Published:** 2024-06-18

**Authors:** Marco Gallo

**Affiliations:** Endocrinology and Metabolic Diseases Unit, Azienda Ospedaliero-Universitaria SS Antonio e Biagio e Cesare Arrigo of Alessandria, 15121 Alessandria, Italy; marco.gallo@ospedale.al.it; Tel.: +39-0131206339; Fax: +39-0131206276

Diabetes, obesity, cardiovascular diseases, and cancer are noncommunicable diseases representing the main global health challenges of the current century.

Although a relationship between diabetes and cancer was first hypothesized more than a century ago [1], this link was only considered as a secondary finding when compared with the relationship between diabetes and cardiovascular diseases, and the topic did not garner mainstream interest for many years. Interestingly, the clinical interest in this link was awakened suddenly, by safety concerns raised by a series of observational reports suggesting a potential association between insulin glargine and tumor risk, in 2009 [2,3].

A long series of observational studies and meta-analyses sometimes confirmed, and many other times denied such an association. In any case, the in-depth study of the topic significantly contributed to conducting increasingly accurate epidemiological analyses from a methodological point of view, and to creating debate regarding the oncological safety of several antidiabetic drugs beyond insulin, from metformin to the newest anti-hyperglycemic, cardiorenal protective agents. To give a few trivial examples, in the period immediately following this, famous joint consensus statements were published [4]; interdisciplinary study groups dedicated to the pathophysiological and clinical study of the relationship between diabetes, obesity, and cancer were established all over the world [5,6]; and several journal special issues dedicated to diabetes and cancer were published, just like the one hosted in this Issue of Cancers.

Mortality rates for cardiovascular disease are decreasing, and the same thing is being observed for mortality rates from several cancers: this extraordinary success is attributable to prevention, early diagnosis, investment in research, and pharmaceutical progress in both of these two big medical fields. In the last decade, “innovative” antidiabetic drugs (incretins and gliflozins) have not only been proven to reduce glycemic targets and to be safe, but also to attain cardiovascular and renal protection to an unpredictable degree. Consequently, there have been significant improvements in diabetes management and declines in rates of both micro- and macrovascular diabetes complications, as well as mortality [7]. At the same time, newer anti-obesity drugs can achieve unprecedented double-digit percentages decreases in initial body weight, with more options to choose from in the coming years. The future for pharmacotherapy of obesity has never been so bright [8].

However, the absolute numbers of people with diabetes and its complications, as well as the numbers of overweight or obese subjects, are constantly growing, also involving low- and middle-income countries of the world in an unprecedented way. Globally speaking, obesity has nearly tripled in the past five decades, and its prevalence is constantly increasing both in adults and children (over 650 million people worldwide). From 1990 to 2022, the combined prevalence of underweight and obesity in adults increased in 162 countries (81%) for women and in 140 countries (70%) for men [9]. The number of people with diabetes, for its part, has quadrupled in the past 30 years and is predicted to rise from current 537 million to 783 million adults by 2045, representing a major challenge for healthcare systems. Furthermore, more than three of every four adults with diabetes live in low- and middle-income countries [10]. This simple and unquestionable observation prompted Venkat Narayan to state, in a renowned speech at the 2015 American Diabetes Association Congress, while receiving the Kelly West Award, that “we are winning the battle but losing the war” [11]. Last but (certainly) not least, the absolute number of people with cancer is constantly increasing. There were about 20 million new cases of cancer in 2022, alongside 9.7 million deaths from cancer worldwide, while the number of new cancer cases is predicted to reach 35 million by 2050 [12].

From a pathophysiological point of view this is not surprising, since obesity, diabetes, cardiovascular diseases, and several types of cancer share most risk factors (e.g., insulin resistance and hyperinsulinemia, chronic inflammation, oxidative stress, lifestyle and voluptuary habits, chronic stress, and so on), thus sketching the mechanistic fundamentals of a ‘dangerous affair’ [13,14]. The Westernization of diet and lifestyle probably played a pivotal role in this process, and emerging evidence shows the additional role played by household and environmental pollution, nanoplastics, and endocrine disruptors too [15,16,17].

Certainly, this absolute increase in the rates of these noncommunicable disease disproportionately impacts on underserved populations and represents a steep challenge for most disadvantaged health systems; it looks like what we could define as the perfect storm is looming on the horizon.

How should we move forward as clinicians, scientific societies, politicians, and stakeholders? I think we are all perfectly aware that there is a PRICE to pay.

“P” stands for prevention, since working to prevent chronic diseases from occurring in the first place is the only sustainable way forward. According to the World Cancer Research Fund, lifestyle changes could prevent 50% of common cancers. However, it is fascinating to note that most of the lifestyle changes recommended to avoid cancer are the same ones we obsessively propose for the prevention of diabetes, metabolic, and cardiovascular diseases: maintaining a healthy weight; moving more; avoiding processed foods and foods rich in sugar or calories; limiting the intake of meat, salt and alcohol; and encouraging breastfeeding [18]. Various vaccinations can prevent the onset of tumors and simultaneously limit the consequences of related infections in obese and/or diabetic subjects. More efforts are needed globally, prioritizing prevention and promoting health equity.

“R” is for research: experimental and translational research should work harder to develop anti-diabetic and weight reduction drugs free from potential mitogenic effects and anti-tumor agents burdened by fewer metabolic and cardiovascular adverse events. It is not science fiction: until just over 10 years ago, diabetologists struggled to do their best using antidiabetic drugs capable of reducing glycated hemoglobin, at the expense of an increased risk of cardiovascular events, heart failure, and mortality. Alongside newer drugs, we need to learn more from basic science, pathophysiology, and epidemiology: What is the role of genetics? What is the role of omic sciences? Can we identify individuals at high risk of developing diabetes or cancer from specific circulating microRNAs?

The “I” stands for interdisciplinary: today, the clinical management of cancer patients with DM or cardiometabolic diseases still relies more on the clinician’s experience than guidelines, representing a major clinical challenge for both oncologists and endocrinologists, just like for hematologists, radiotherapists, cardiologists, and palliativists. Cancer management has progressed significantly in previous decades, requiring a multidisciplinary team approach to provide the best possible patient care, and to cope with the various comorbidities, complications, and adverse events arising during the patient’s treatment journey. New interdisciplinary disciplines such as cardio-oncology, onco-nephrology, and diabeto-oncology are emerging to ensure the best possible care for oncologic patients based on a comprehensive approach [19].

“C” stands for community policies. Communities and healthcare systems must interact, through policy and the active participation of citizens in public decision-making processes, to obtain healthier environments in which people can be conceived, grow, work, live, and age. What is the impact on diabetes and/or cancer of long-term exposure to fine particulate matter, or to endocrine disruptors? It is possible to create a better world to live in through effective laws and regulations, as well as the allocation of resources. The WHO initiative “One Health” sustains an integrated, unifying approach that aims to sustainably balance and optimize the health of people, animals, and ecosystems, recognizing that the health of people and all other living beings are closely interconnected [20]. Another virtuous initiative is represented by Cities Changing Diabetes, an international program addressing the prevention and management of type 2 diabetes in urban settings in major cities around the world, emphasizing participation and co-creation of citizens and professional stakeholders in research and development for social change, health promotion, and diabetes prevention [21]. Other government policy choices can also make a difference in reducing the impact of this perfect storm, such as price control on tobacco, alcohol, and taxing high-sugar or high-fat foods.

Last but not least, we all need more “Education”: the time has come for scientific societies and academic centers to train ad hoc diabetologists, endocrinologists, internists, and cardiologists who practice in the oncology field, and hematologists/oncologists who really care about cancer-related metabolic and cardiovascular issues.

This is definitely a steep PRICE: are we all really persuaded that we will have to pay for it?
